# Pharmacogenes that demonstrate high association evidence according to CPIC, DPWG, and PharmGKB

**DOI:** 10.3389/fmed.2022.1001876

**Published:** 2022-10-25

**Authors:** Mohammad A. Alshabeeb, Mesnad Alyabsi, Mohammad A. Aziz, Salah Abohelaika

**Affiliations:** ^1^Population Health Research Section, King Abdullah International Medical Research Center, Riyadh, Saudi Arabia; ^2^King Saud Bin Abdulaziz University for Health Sciences, Ministry of National Guard Health Affairs (MNGHA), Riyadh, Saudi Arabia; ^3^Interdisciplinary Nanotechnology Centre, Aligarh Muslim University, Aligarh, India; ^4^Department of Pharmacy, Qatif Central Hospital, Qatif, Saudi Arabia

**Keywords:** pharmacogenes, CPIC, DPWG, PharmGKB, evidence levels, gene panel, genetic testing

## Abstract

**Background:**

Different levels of evidence related to the variable responses of individuals to drug treatment have been reported in various pharmacogenomic (PGx) databases. Identification of gene-drug pairs with strong association evidence can be helpful in prioritizing the implementation of PGx guidelines and focusing on a gene panel. This study aimed to determine the pharmacogenes with the highest evidence-based association and to indicate their involvement in drug-gene interactions.

**Methodology:**

The publicly available datasets CPIC, DPWG, and PharmGKB were selected to determine the pharmacogenes with the highest drug outcome associations. The upper two levels of evidence rated by the three scoring methods were specified (levels A–B in CPIC, 3–4 in DPWG, or 1–2 levels in PharmGKB). The identified pharmacogenes were further ranked in this study based on the number of medications they interacted with.

**Results:**

Fifty pharmacogenes, with high to moderately high evidence of associations with drug response alterations, with potential influence on the therapeutic and/or toxicity outcomes of 152 drugs were identified. CYP2D6, CYP2C9, CYP2C19, G6PD, HLA-B, SLCO1B1, CACNA1S, RYR1, MT-RNR1, and IFNL4 are the top 10 pharmacogenes, where each is predicted to impact patients' responses to ≥5 drugs.

**Conclusion:**

This study identified the most important pharmacogenes based on the highest-ranked association evidence and their frequency of involvement in affecting multiple drugs. The obtained data is useful for customizing a gene panel for PGx testing. Identifying the strength of scientific evidence supporting drug-gene interactions aids drug prescribers in making the best clinical decision.

## Introduction

Multiple mutations located in pharmacogenes which encode proteins (e.g., metabolizing enzymes and drug transporters) (1) may affect drug disposition, resulting in differences in drug pharmacology (2, 3). Hence, pharmacogenomic (PGx) testing is a very useful approach for individualizing patient therapies and predicting their variable responses to drugs according to their genotype (4, 5). Many patients have poor drug outcomes; for example, 30–60% of patients taking beta-2 adrenergic agonists (antiasthmatics), 10–30% of patients taking angiotensin-converting enzyme (ACE) inhibitors, 15–25% of patients taking beta blockers, and 30–70% of patients taking statins do not respond well to their drug therapy (6). In addition, adverse drug reactions (ADRs) are a major problem that may lead to higher rates of hospitalization and increased morbidity, resulting in a significant strain on healthcare systems (7, 8). These may be partially avoided by screening the genetic backgrounds of patients and identifying the people at risk.

Currently, several clinical guidelines and PGx recommendations, such as the 73 recommendations developed by the international Clinical Pharmacogenetics Implementation Consortium (CPIC; https://cpicpgx.org/) (9, 10) and the 63 recommendations suggested by the Dutch Pharmacogenetics Working Group (DPWG) established by the Royal Dutch Pharmacist's Association (KNMP; https://www.knmp.nl/) (11), are routinely used in multiple advanced medical centers in Western countries (12). In addition, the Pharmacogenomics Knowledge Base (PharmGKB; www.pharmgkb.org) (13), established by Stanford University and funded by the National Institutes of Health (NIH), is a large comprehensive pharmacogenomics dataset that annotates existing clinical practice guidelines (14, 15). The guidelines provide clear advice to change, avoid, or monitor drug therapies based on patients' unique genotypes; such genotypes that require changes in the treatment plan are known as actionable genotypes (16).

The existing scientific evidence has driven a number of worldwide regulatory agencies, in particular, the United States Food and Drug Administration (USFDA) (17), the American College of Medical Genetics and Genomics (ACMG) (18), the European Medicine Agency (EMA) (19), The French National Network of Pharmacogenetics (RNPGx) (20), the Pharmaceuticals and Medical Devices Agency (PMDA) in Japan (21), the Canadian Pharmacogenomics Network for Drug Safety (CPNDS) (22), and the Canadian Health Agency [Health Canada (Santé Canada) (HCSC)] (22), to be in favor of selecting particular genetic tests prior to the administration of pharmacogenetic drugs. However, the selected drugs to test for and the PGx information described in the drug labels vary between different health agencies based on their variable assessment of the evidence level for gene-drug association and actionability (23, 24). For example, as of September 14, 2022, the number of drugs where genetic testing is required or recommended prior to drug use by FDA, EMA, HCSC, and PMDA is 127, 75, 45, and 15, respectively, as assessed by PharmGKB curators and add the refernce number (13). Currently, several hundred of genes and thousands of genetic markers are described in various PGx databases. Therefore, this study aimed to identify the most important pharmacogenes with robust association evidence to facilitate the implementation of the relevant clinical guidelines.

## Methodology

This study focused on pharmacogenes with well-established drug response associations according to the evidence shown in three reliable scoring methods: CPIC, DPWG and PharmGKB consortia. CPIC and DPWG databases which focus on the clinical actionability of gene/drug pairs were selected based on certain criteria: (i) they rank associations between genes and drug outcomes based on peer-reviewed publications, (ii) all possible genes are included, (iii) focus on clinical utility of PGx tests and provide gene–drug dosing guidelines, (iv) the most reliable PGx databases in the US and Europe. In addition, PharmGKB was selected as a scoring database as it (i) provides evidence levels ranking too based on clinical annotations reported in the curated literature (25). Thus, PharmGKB is considered the preeminent comprehensive pharmacogenomic global database which assesses the clinical validity of PGx data reported in the literature. Other PGx useful databases are also available but were not selected in our study as no scoring system encompassed and some resources were designed to cover limited genes. For example, Helix PGx database (https://github.com/myhelix/helix-pgxdb) provides allelic information related to four genes only (CYP2C9, CYP2C19, CYP2D6, and CYP4F2), while PharmVar database was designed to cover PGx data for cytochrome P450 genes but recently three more non-CYP genes were introduced (NUDT15, SLCO1B1, and DPYD). Similarly, the Canadian Pharmacogenomics Network for Drug Safety (CPNDS) was not considered as it is focused only on genetic markers relevant to severe and rare adverse drug reactions (26). The French database (RNPGx) provides PGx guidelines and genetic testing recommendations but was not selected in this study as the data are not publicly available and the used level of evidence was assessed based on the functionality of variants rather than relying on the quality of supporting literature. In contrast, the selected publicly available databases in our study were screened to identify the most important pharmacogenes of interest based on strong scientific evidence. Selection of gene panel in our approach was not limited to a specific drug category.

As of September 14, 2022, the existing association pairs (gene/drug effect) reported by the CPIC reached 448 associations between 271 drugs and 119 genes (9). A much larger number of genes (*n* = 1,803) are described in the PharmGKB database affecting 747 drugs (13). In contrast, limited genes (*n* = 13) and drugs (*n* = 53) have been reported by DPWG (11). In our study, only the upper two levels of evidence ranked with high to moderately high association gene/drug pairs (those classified as A-B levels by CPIC, level 3–4 by DPWG, or 1–2 levels by PharmGKB) were included. Other pairs with lower association significance were excluded because of inadequate evidence of actionability. The levels of evidence described by CPIC and PharmGKB were identified directly from the searching engine provided by CPIC website (https://cpicpgx.org/genes-drugs/). The used searching terms included the PharmGKB evidence levels (1A, 1B, 2A, and 2B) which enabled us to identify all ranked drug-gene pairs under these levels. The obtained results were further confirmed through a separate search in the PharmGKB searching engine (https://www.pharmgkb.org/). The full table of CPIC level status can be downloaded as an excel sheet where filtering is possible to restrict the list of A and B levels. In addition, the gene-drug pairs suggested by DPWG (level 3–4) was screened manually through reviewing the latest PGx updated report (01/02/2022, https://www.knmp.nl/media/1058). The definitions of the different levels of evidence related to the three selected consortia are presented in Table 1. The extent of interaction of each shortlisted gene with various medications was investigated to highlight the major genes with higher involvement in drug-gene interactions. The study was approved by the Institutional Review Board (IRB) at King Abdullah International Medical Research Center (KAIMRC) (NRC21R/394/09).

**Table 1:** Definitions of different levels of gene-drug association evidence.

**Levels of evidence**	**DPWG**	**CPIC**	**PharmGKB**
High	**4 =** Published controlled studies of **good quality** and having relevant pharmacokinetic or clinical endpoints.	**A =** Strong or moderate recommendations which **should** be used to change prescribing of affected drug.	**1A =** The association is **endorsed by a medical society into PGx guideline, or implemented** at a major health system.
			**1B =** The association must be **replicated in more than one cohort** with significant *p* values, and preferably have a strong effect size.
Moderately high	**3 =** Published controlled studies **of moderate quality** and having relevant pharmacokinetic or clinical endpoints.	**B =** Moderate recommendations which **could** be used to change prescribing of the affected drug because alternative therapies/dosing are extremely likely to be as effective and as safe as non-genetically based dosing.	**2A =** The association with a **variant located within a VIP (Very Important Pharmacogene)**. So functional significance is more likely **2B =** The association must be **replicated** but there may be **some studies that do not show statistical significance**, and/or **the effect size may be small**.
Moderately low	**2 =** Published **case reports** and well documented **case series**.	**C =** There are published studies at varying levels of evidence, some with mechanistic rationale, **but no prescribing actions** are recommended.	**3 =** An association based on a **single significant (not yet replicated)** study or evaluated in multiple studies but **lacking clear evidence** of an association.
Low	**1 =** Published **incomplete case reports** and Product information.	**D =** There are **few** published studies, clinical actions are **unclear**.	**4 = Case report, non-significant study or in vitro**, molecular or functional assay.

## Results

### Genes which met the evidence criteria reported by CPIC, DPWG, and PharmGKB

**Figure 1:**
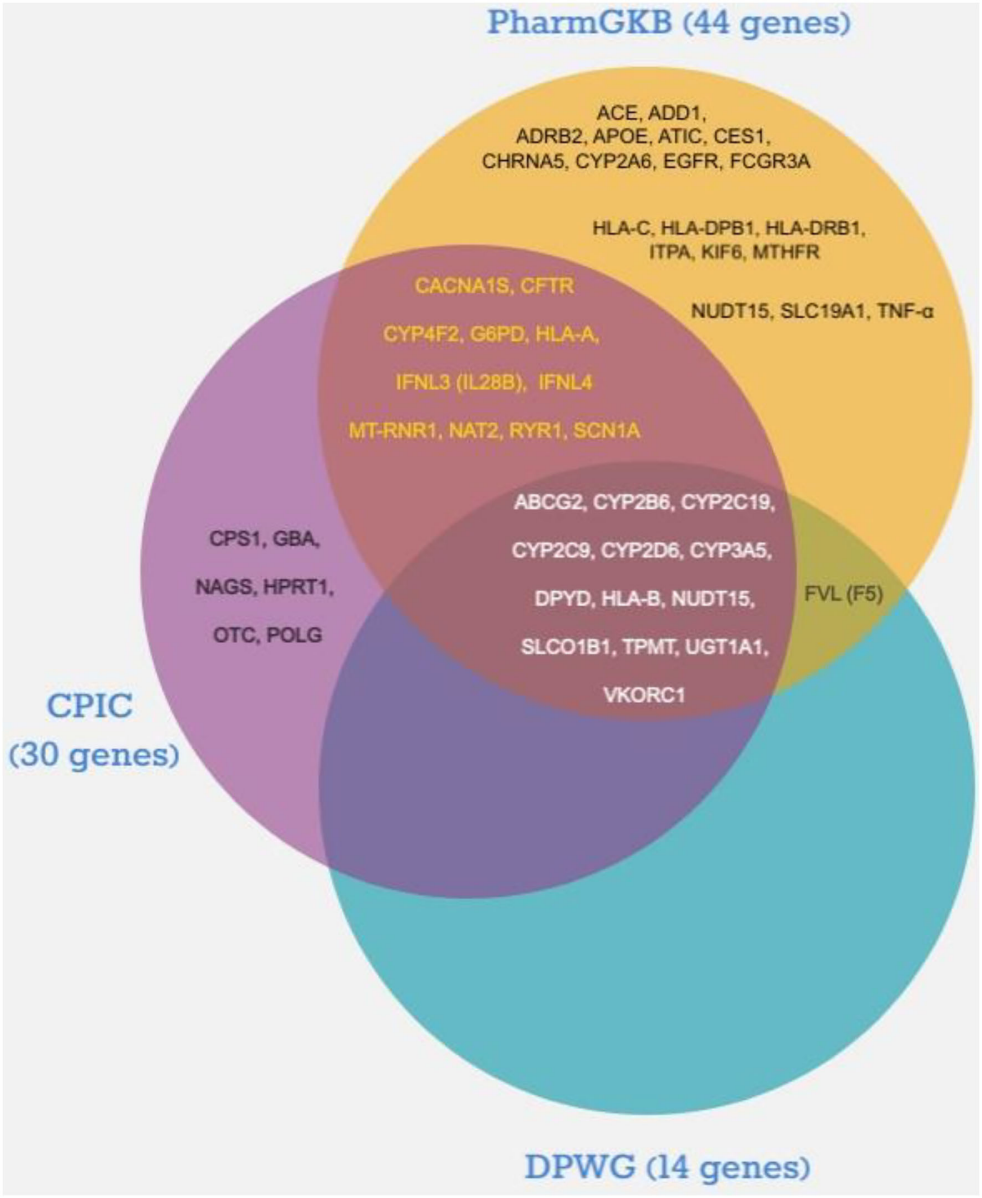
Distribution of pharmacogenes with highest association evidence according to the Dutch Pharmacogenetics Working Group (DPWG), the Clinical Pharmacogenetics Implementation Consortium (CPIC), and Pharmacogenomics Knowledge Base (PharmGKB).

As illustrated in Figure 1, the number of pharmacogenes that fulfilled the inclusion criteria and met the indicated levels of association evidence according to CPIC, DPWG, and PharmGKB scoring was 30, 14, and 44, respectively. Thirteen of the pharmacogenes (ABCG2, CYP2B6, CYP2C19, CYP2C9, CYP2D6, CYP3A5, DPYD, HLA-B, NUDT15, SLCO1B1, TPMT, UGT1A1, and VKORC1) labeled by DPWG were also considered by both CPIC and PharmGKB. In contrast, another 11 genes [CACNA1S, CFTR, CYP4F2, G6PD, HLA-A, IFNL3 (IL28B), IFNL4, MT-RNR1, NAT2, RYR1, and SCN1A] were reported only by CPIC and PharmGKB. In addition, the Factor V Leiden (FVL or F5) gene was only suggested by DPWG and PharmGKB among the list supported by high evidence. Six genes (CPS1, GBA, HPRT1, NAGS, OTC, and POLG) were recommended by CPIC only, whereas the remaining 19 evidently supported genes are also appeared in the PharmGKB list. Of the 44 pharmacogenes reported in the PharmGKB database with strong evidence, 28 genes were described by PharmGKB as very important pharmacogenes (VIP). The VIP genes listed by PharmGKB are classified into three categories based on the seriousness of drug outcome regardless of evidence levels: Tier 1 (genes with considerable evidence), Tier 2 (genes with limited evidence), and cancer genome (genes affecting anticancer efficacy and toxicity) (*n* = 34, 25, and 9 VIPs, respectively) (13). Among the Tier 1 VIP genes, 27 (79.4%) fit the evidence levels chosen in this study. The remaining Tier 1 genes (*n* = 7) with low association evidence, which were excluded, were ABCB1, ADRB1, COMT, CYP2C8, DRD2, GSTP1, and TYMS. In contrast, only one VIP gene in the Cancer Genome group (EGFR) met the evidence criteria, but none of the Tier 2 genes were included. The total number of unique pharmacogenes that met the top two highest rankings of evidence by the three selected PGx consortia was 50 (Table 2); descriptions of their names, gene ontology, and biological functions are shown in the Supplementary Table.

**Table 2:** Pharmacogenes with satisfactory evidence of drug response associations based on CPIC, PharmGKB and DPWG.

**Genes** **(*n* = 50)**	**Alleles/SNPs**	**Interacting drugs**	**Total no. of drugs**
ABCG2	rs2231142 (T)	Allopurinol, Rosuvastatin	2
ACE	rs1799752 (del)	Captopril	1
ADD1	rs4961 (T)	Hydrochlorothiazide	1
ADRB2	rs1042713 (A)	Salmeterol	1
APOE	rs7412 (C)	Atorvastatin	1
ATIC	rs4673993 (T)	Methotrexate	1
CACNA1S	rs772226819 (A), rs1800559 (T)	Desflurane, Enflurane, Halothane, Isoflurane, Methoxyflurane, Sevoflurane, Succinylcholine	7
CES1	rs71647871 (A)	Clopidogrel	1
CFTR	34 SNPs	Ivacaftor	1
CHRNA5	rs16969968 (A)	Nicotine	1
CPS1	rs1047891 (A)	Valproic acid	1
CYP2A6	*2, *4A, *7, *9A, *10, *12, *17, *19, *20, *24A, *26, *27, *28A	Nicotine	1
CYP2B6	*4 [rs2279343 (G)], *6 [rs3745274 (T)+ rs2279343 (G)]	Bupropion, Methadone	4
	rs3745274 (T), *6, rs28399499 (C), *18 [rs3745274 (T) + rs28399499 (C)], *26 [*6+rs3826711 (G)]	Efavirenz	
	rs3745274 (T), rs28399499 (C)	Nevirapine	
CYP2C9	rs1799853 (T, *2), rs1057910 (C, *3)	Acenocoumarol, Celecoxib, Flurbiprofen, fluvastatin, Fosphenytoin, Ibuprofen, Lornoxicam, Meloxicam, Phenytoin, Piroxicam, Siponimod, Tenoxicam, Warfarin	13
CYP2C19	rs4244285 (A, *2), rs4986893 (A, *3), rs12248560 (T, *17)	Amitriptyline, Citalopram, Clomipramine, Clopidogrel, Dexlansoprazole, Doxepin, Escitalopram, Imipramine, Lansoprazole, Brivaracetam, Omeprazole, Pantoprazole, Rabeprazole, Sertraline, Trimipramine, Voriconazole	16
CYP2D6	*3, *4, *5, *6, *9, *10, *17, *29 *41 or duplication	Amitriptyline, Aripiprazole, Atomoxetine, Clomipramine, Codeine, Desipramine, Doxepin, Eliglustat, Flecainide, Fluvoxamine, Haloperidol, Hydrocodone, Imipramine, Metoprolol, Mirtazapine, Nortriptyline, Oliceridine, Ondansetron, Oxycodone, Paroxetine, Pimozide, Pitolisant, Propafenon, Risperidone, Tamoxifen, Tetrabenazine, Tramadol, Trimipramine, Tropisetron, Venlafaxine, Vortioxetine, Zuclopenthixol	32
CYP3A4	rs28371759 (G, *18), rs2242480 (T, *36)	Fentanyl	3
	rs67666821 (TT, *20), rs35599367 (A, *22),	Quetiapine	
	rs4646437 (A), rs4986910 (G, *3), rs28371759 (G, *18), rs67666821 (TT, *20), rs35599367 (A, *22), rs2242480 (T, *36)	Tacrolimus	
CYP3A5	rs776746 (A, *1)	Tacrolimus	1
CYP4F2	rs2108622 (T, *3)	Acenocoumarol, Phenprocoumon, Warfarin	3
DPYD	rs3918290 (A, *2A), rs55886062 (G, *13), rs56038477 (A), rs67376798 (T)	Capecitabine, Fluorouracil, Tegafur	3
EGFR	rs121434568 (T), rs121434569 (C)	Gefitinib	2
	rs121434569 (C)	Erlotinib	
FCGR3A	rs396991 (A)	Rituximab	1
FVL	rs6025 (A)	Contraceptives with Estrogen	1
GBA	48 SNPs [e.g. rs76763715 (C), rs79653797 (G)]	Velaglucerase alfa	1
G6PD	68 SNPs [e.g. rs1050829 (G), rs1050828 (A), rs5030868 (T)]	Aspirin, Chloramphenicol, Chlorpropamide, Ciprofloxacin, Dapsone, Dimercaprol, Glibenclamide, Glimepiride, Glipizide, Mafenide, Mesalazine, Methylene blue, Moxifloxacin, Nalidixic acid, Nitrofurantoin, Norfloxacin, Pegloticase, Phenazopyridine, Primaquine, Probenecid, Quinine, Rasburicase, Sodium nitrite, Sulfacetamide, Sulfadiazine, Sulfamethoxazole / Trimethoprim, Sulfasalazine, Sulfisoxazole, Tafenoquine	29
HLA-A	*31:01	Carbamazepine	2
	*33:03	Allopurinol	
HLA-B	*13:01	Dapsone	10
	*15:02	Carbamazepine, Fosphenytoin, Lamotrigine, Oxcarbazepine, Phenytoin	
	*15:11	Carbamazepine	
	*57:01	Abacavir, Flucloxacillin	
	*58:01	Allopurinol	
	*59:01	Methazolamide	
HLA-C	*03:01	Allopurinol	3
	*01:02	Methazolamide	
	*04:01	Nevirapine	
HLA- DPB1	*02:01	Aspirin	1
HLA-DRB1	*01:01	Nevirapine	1
HPRT1	del	Mycophenolic acid	1
IFNL3 (IL28B)	rs11881222 (rs368234815, G)	Peginterferon Alfa-2a, Peginterferon Alfa-2b, Ribavirin	3
IFNL4	rs12979860 (T), rs11322783 (G), rs8099917 (G)	Boceprevir, Peginterferon Alpha-2a, Peginterferon Alpha-2b, Ribavirin, Telaprevir	5
ITPA	rs1127354 (C), rs7270101 (C)	Peginterferon Alpha-2b, Ribavirin	2
KIF6	rs20455 (C)	Pravastatin	1
MTHFR	rs1801133 (G)	Methotrexate	1
MT-RNR1	rs267606617 (G), rs267606619 (T)	Amikacin, Gentamicin, Kanamycin, Paromomycin, Plazomicin, Streptomycin, Tobramycin	7
NAGS	29 SNPs	Carglumic acid, Valproic acid	2
NAT2	4 slow acetylator SNPs (*5, *6, *7, 14*), 3 rapid or intermediate acetylators (*4, *12, *13)	Hydralazine, Isoniazid	2
NUDT15	rs116855232 (T)	Azathioprine, Mercaptopurine, Thioguanine	3
OTC	rs72554356 (T)	Valproic acid	1
POLG	rs2307441 (C)	Divalproex sodium, Valproic acid	2
RYR1	44 SNPs [e.g. rs111888148 (A), rs112563513 (A)]	Desflurane, Enflurane, Halothane, Isoflurane, Methoxyflurane, Sevoflurane, Succinylcholine	7
SCN1A	rs3812718 (T)	Carbamazepine, Phenytoin	2
SLC19A1	rs1051266 (C)	Methotrexate	1
SLCO1B1	rs4149056 (*5, C), rs2306283 (*1B, G)	Atorvastatin, Fluvastatin, Lovastatin, Pitavastatin, Pravastatin, Rosuvastatin, Simvastatin	7
TNF-α	rs1800629 (A)	Etanercept	1
TPMT	*2, *3A, *3B, *3C	Azathioprine, Mercaptopurine, Thioguanine	3
UGT1A1	rs8175347 [(TA)7R, *28]	Atazanavir, Belinostat, Irinotecan, SN-38	4
VKORC1	rs9923231 (A, *2), rs7294 (C, *3), rs9934438 (A)	Warfarin	1

*Star allele nomenclature given to identify genetic variants.

### Medications affected by the suggested genes

The 50 genes indicated in Table 2 have the potential to impact 152 therapeutic drugs listed in Table 3. Cytochrome P450 genes (CYP2D6, CYP2C19, and CYP2C9) were identified among the top five genes affecting the outcomes of a larger number of medications (*n* = 32, 16, and 13, respectively) (Table 2). Furthermore, G6PD and HLA-B, which came in second and fifth, respectively, were associated with variable phenotypes related to 29 and 10 drugs, respectively. The other top-ranked genes include CACNA1S, MT-RNR1, RYR1, and SLCO1B1 where each interacts with seven medications, in addition to IFNL4 which may interact with five medications.

**Table 3:** List of medications predicted to be impacted by the 50 pharmacogenes.

**PGx drugs (*n* = 152)**
Abacavir	Dimercaprol	Irinotecan	Ondansetron	Sertraline
Acenocoumarol	Divalproex sodium	Isoflurane	Oxcarbazepine	Sevoflurane
Allopurinol	Doxepin	Isoniazid	Oxycodone	Simvastatin
Amikacin	Efavirenz	Ivacaftor	Pantoprazole	Siponimod
Amitriptyline	Eliglustat	Kanamycin	Paromomycin	SN-38
Aripiprazole	Enflurane	Lamotrigine	Paroxetine	Sodium nitrite
Aspirin	Erlotinib	Lansoprazole	Peginterferon Alfa-2a	Streptomycin, Succinylcholine
Atazanavir	Estradiol containing contraceptives	Lornoxicam	Peginterferon Alfa-2b	Sulfacetamide, Sulfadiazine
Atomoxetine	Escitalopram	Lovastatin	Pegloticase	Sulfamethoxazole/Trimethoprim
Atorvastatin	Etanercept	Mafenide	Phenazopyridine	Sulfasalazine
Azathioprine	Fentanyl	Meloxicam	Phenprocoumon	Sulfisoxazole
Belinostat	Flecainide	Mercaptopurine	Phenytoin	Tacrolimus
Brivaracetam	Flucloxacillin	Mesalazine	Pimozide	Tafenoquine
Bupropion	Fluorouracil	Methadone	Piroxicam	Tamoxifen
Capecitabine	Flurbiprofen	Methazolamide	Pitavastatin	Tegafur
Captopril	Fluvastatin	Methotrexate	Pitolisant	Telaprevir
Carbamazepine	Fluvoxamine	Methoxyflurane	Plazomicin	Tenoxicam
Carglumic acid	Fosphenytoin	Methylene blue	Pravastatin	Tetrabenazine
Celecoxib	Gefitinib	Metoprolol	Primaquine	Thioguanine
Chloramphenicol	Gentamicin	Mirtazapine	Probenecid	Tobramycin
Chlorpropamide	Glibenclamide	Moxifloxacin	Propafenone	Tramadol
Ciprofloxacin	Glimepiride	Mycophenolic acid	Quetiapine	Trimipramine
Citalopram	Glipizide	Nalidixic acid	Quinine	Tropisetron
Clomipramine	Haloperidol	Nevirapine	Rabeprazole	Valproic acid
Clopidogrel	Halothane	Nicotine	Rasburicase	Velaglucerase alfa
Codeine	Hydralazine	Nitrofurantoin	Ribavirin	Venlafaxine
Dapsone	Hydrochlorothiazide	Norfloxacin	Rituximab	Voriconazole
Desflurane	Hydrocodone	Nortriptyline	Risperidone	Vortioxetine
Desipramine	Ibuprofen	Oliceridine	Rosuvastatin	Warfarin
Dexlansoprazole	Imipramine	Omeprazole	Salmeterol	Zuclopenthixol

## Discussion

The large pharmacogenomic information and its complexity make it difficult for healthcare providers to implement PGx guidelines (27, 28). Therefore, prioritizing the PGx recommendations and focusing on the more important genes affecting drug responses based on the level of evidence reported by distinctive PGx databases can be supportive for physicians in making appropriate clinical decisions (29). Hence, this research attempted to narrow the extensive repository of data and provides a practical evidence-based approach that suggests a panel of genes for clinical preemptive testing. The suggested panel described multiple variants in 50 genes that potentially affected 152 drugs based on high the scientific evidence indicated collectively in the CPIC, DPWG, and PharmGKB. However, several hundred gene/drug pairs with lower associations can be reassessed and possibly added to the suggested panel in the future as more information and/or stronger evidence become available.

Designing a selected gene panel to explore precise genomic sequences of interest is a unique approach to minimize genetic testing costs, decreases the burden of analyzing big data, and gives more chances to examine a larger number of samples (30). Our suggested PGx panel is novel as it covers broad drugs from various therapeutic areas and encompasses 100% of genes rated among the upper two levels of evidence described in the three selected databases. Some genes (*n* = 40) which were classified by PharmGKB as VIP based on their functions were excluded from our suggested gene list as they failed to meet the evidence criteria. Unlike our panel, several PGx gene panels are commercially available but full coverage of genes with high evidence is lacking. For example, coverage of PharmGKB genes (ranked as 1A, 1B, 2A, and 2B) by six widely used commercial panels is unsatisfactory (22) and ME [1A (19%), 1B (27%), 2A (32%), 2B (32%)], Living DNA [1A (60%), 1B (64%), 2A (58%), 2B (59%)], PharmacoScan [1A (63%), 1B (68%), 2A (65%), 2B (70%)], DMET Plus [1A (44%), 1B (36%), 2A (45%), 2B (11%)], Ion AmpliSeq Pharmacogenomics [1A (37%), 1B (36%), 2A 35%), 2B (8%)], and IPLEX PGx Pro [1A (38%), 1B (32%), 2A (38%), and 2B (5%)] (31). Similarly, coverage of 19 CPIC genes with strong evidence in the six largest PGx panels available in the US was also incomplete [Drug Response Panel (84%), PGXONE Plus (74%), PGx Complete (63%), PharmacoDx (53%), RightMed (79%), and Polypharmacy Comprehensive Panel (74%)] (32).

Recently, the ClinGen database, founded by the National Human Genome Research Institute in the United States (US) proposed a list of pharmacogenes with high clinical relevance (33). In contrast to our study, ClinGen suggested a higher number of genes (*n* = 127) based on wider levels of association evidence reported in CPIC (Levels A–D) and PharmGKB (Levels A and B). In our study, we used more consistent criteria by restricting the list to drug-gene associations reported among the upper two levels of evidence only of all involved databases; therefore, we excluded levels C and D of the CPIC. In addition to the CPIC and PharmGKB, commonly used PGx databases in the US, we suggested the nomination of the genes listed by DPWG, which is the most accepted consortium used in Europe, although all its list of genes (*n* = 13) are already included in the

other two selected databases (CPIC and PharmGKB). Focusing on drug-gene associations that are ranked among the top two level of evidence may become a trend in PGx research. Several recent studies implemented this approach; for example, one study assessed the genetic coverage of six various PGx assays. These assays were compared to each other to determine the best assay with uppermost coverage of genes with highest evidence focusing on level 1 A–B and 2 A–B genes listed by PharmGKB (31). Another study screened 293 genes among Slovenians to identify their potential pharmacogenetic characteristics, however, they focused only on 85 variants with association evidence level of PharmGKB 1A or 1B level (*n* = 24 variants) and level 2A or 2B (61 variants) (34). Very recently, 1116 Hong Kong Chinese subjects were investigated to assess frequency of 133 PGx variants with highest level of evidence according to PharmGKB (35). The databases we used generated their PGx data from various populations with different ethnicities, thus, our suggested gene-panel can be customized to suit the unique genetic make-up of a selected population.

The data obtained in our study clearly showed that CYP2D6 is the most important gene because of the potential probability of metabolizing 32 clinically used drugs as supported by strong evidence. CYP2D6, located on chromosome 22 and expressed mainly in the liver, plays a major role in the elimination of several xenobiotic molecules and consumed chemicals in addition to its primary function in drug metabolism (36). Patients carrying CYP2D6 variants with decreased activity, such as the ^*^9 and ^*^10 alleles, or carrying inactive alleles such as the ^*^3-^*^8 show low gene function, moderate or poor drug metabolization (37). The intake of prodrugs, for example, codeine, requires satisfactory metabolism by CYP2D6 to be converted into its active substance (morphine). Thus, patients with slow metabolizing phenotypes may fail to achieve adequate analgesia and are predicted to develop more adverse reactions related to the accumulation of codeine (38). In this case, alternative drugs (non-CYP2D6 substrates), such as paracetamol, non-steroidal anti-inflammatory drugs (NSAIDs), or morphine, are recommended (39).

In our study, other cytochrome P450 genes, such as CYP2C9 and CYP2C19, located on chromosome 10, were also emphasized by the three selected PGx databases among the pharmacogenes that affect the metabolism of large numbers of drugs. Cytochrome P450, in particular CYP2D6, CYP2C9, and CYP2C19, are commonly tested in clinical practice, and their roles are well understood (16). Analysis of these genes prior to the administration of relevant drugs aims to optimize their therapeutic outcomes (40). For instance, serious incidents of arterial thrombosis may develop as a result of therapy failure of clopidogrel, a pro-drug platelet inhibitor, which requires CYP2C19 metabolism to be converted to its active drug metabolite. Clopidogrel resistance was frequently seen in patients positive for ^*^2 and ^*^3 alleles in CYP2C19 (41). Likewise, multiple cases of therapy resistance and thromboembolism or high level of sensitivity and bleeding related to the anticoagulant agent, warfarin, were reported, associated with variable risk variants observed in CYP2C9 (42, 43). Patients with ^*^2 and/or ^*^3 mutations in CYP2C9 are slower metabolizers of warfarin by 10% approximately (44), which makes a significant impact on warfarin accumulation in blood as a result of its narrow therapeutic index.

The glucose-6-phosphate dehydrogenase (G6PD) gene came next to CYP2D6 in terms of the number of affected drugs (*n* = 29). Individuals with enzymatic deficiency of G6PD, with mutations on the X chromosome, are generally asymptomatic; however, they may experience acute hemolytic anemia upon exposure to the indicated medications, in particular sulfa drugs, and some foods such as fava beans (45). Preemptive genetic testing can help prevent such incidents, which can be fatal unless treated early and properly (46). Although various quantitative spectrophotometric assays are available to measure G6PD activity, enzyme deficiency can be masked in the case of marked reticulocytosis, a heterozygous female, a very high white blood cell count, and in recent blood transfusions (47). Thus, molecular testing should be considered. A wide range of polymorphisms in the human leukocyte antigen (HLA)-B loci, located in the major histocompatibility complex (MHC) region on chromosome 6, have been shown to be risk factors for various adverse reactions (e.g., hypersensitivity and hepatotoxicity) related to nine different medications (48, 49). HLA-B^*^57:01 is a famous example of an allele seriously associated with life-threatening reactions as a consequence of exposure to abacavir (induces severe hypersensitivity) (50) and flucloxacillin (induces liver injury) (51). In addition, testing for various variants in HLA-A, HLA-B, and HLA-C is helpful in predicting patients at risk of developing severe cutaneous reactions induced by several drugs, such as carbamazepine, allopurinol (52), and lamotrigine (53). The solute carrier organic anion transporting polypeptide 1B1 (SLCO1B1) gene, located on chromosome 12, encodes organic anion transporting polypeptide 1B1 (OATP1B1), which acts as a drug uptake transporter. The 1B1^*^15 haplotype, which includes SLCO1B1^*^5 (rs4149056, 521T > C) and SLCO1B1^*^1B (rs2306283, 388 A > G), has been identified as a risk factor for myopathy caused by multiple statins (*n* = 7); lipid-lowering agents (54). This allele may possibly affect methotrexate-related toxicity, though the association evidence level is moderately low (55).

This study revealed other important pharmacogenes such as ryanodine receptor 1 (RYR1), calcium voltage-gated channel subunit alpha1 S (CACNA1S), and mitochondrially encoded 12S RNA (MT-RNR1), which also interact with several drugs (*n* = 7) and possibly impact patient outcomes. Numerous polymorphisms in RYR1 and CACNA1S, which encode the ryanodine receptor and calcium channel in skeletal muscles, respectively, on chromosomes 19 and 1 (56), are associated with malignant hyperthermia when patients are preoperatively exposed to one of the anesthetic agents from the flurane group (desflurane, enflurane, isoflurane, methoxyflurane, sevoflurane), halothane, or succinylcholine (57, 58). Nephrotoxicity and ototoxicity induced by aminoglycosides (amikacin, gentamicin, kanamycin, paromomycin, streptomycin, and tobramycin) are possibly related to variants in MT-RNR1, the mitochondrial RNA gene (59).

Patient responses to five antiviral agents (boceprevir, peginterferon alfa-2a, peginterferon alfa-2b, ribavirin, and telaprevir) are predicted to be influenced by the intronic variant rs12979860 located in the interferon lambda 4 (IFNL4) gene. This gene, located on chromosome 19, encodes a protein (cytokine) with ligands that form a complex through binding with IFN lambda receptors, which results in activation of the Janus kinase-signal transducer and activator of transcription (JAK-STAT) signaling and upregulation of multiple interferon genes. This pathway mediates ultimately a major role in protection from viral infection, particularly hepatitis C virus (HCV) (60). Patients with the CC genotype of rs12979860 showed higher viral clearance than the TT genotype in response to treatment with peginterferon alpha-2a or 2b in addition to ribavirin (61). Response to these three medications may also be influenced by other variants that possibly impact the function of interferon genes [rs11322783 (formerly known as rs368234815); exonic variant in IFNL4, rs8099917; intronic variant located upstream of IFNL4, and rs11881222; intronic variant in IFNL3] (62, 63). Genetic testing of patients using the indicated antiviral agents may be useful to ensure optimal therapeutic efficacy. Alternatively, the new hepatitis C therapies such as sofosbuvir, ledipasvir, and daclatasvir, which are not known to be affected by the mutations in IFNL3 and IFNL4 genes, can be used (64). However, it has been reported that selected polymorphisms in the ABCB1 and HNF-α genes influence the concentration of sofosbuvir plasma metabolite (65).

The ranking of genes in this study was made based on the level of association evidence and the number of drugs affected by each gene. In addition, identifying genes that affect the most commonly prescribed medications has to be considered as a criterion for selecting panel genes (66). In a previous study which investigated usage of PGx drugs over 7 years in the Netherlands, CYP2D6, SLCO1B1, and CYP2C19 were only found to be influencing 95% of the PGx drugs used by 11.4 million individuals (67). According to our study criteria, the three genes were nominated among the top-ranked genes.

Educating drug prescribers about the precautions of PGx medications, particularly the drugs identified in this study, and the benefits of ordering genetic testing may attract them to the concept of personalized medicine versus the usual standard of care (68). The evolution of practicing medicine has gone from empirical to evidence-based. Evidence-based medicine provides the necessary information that allows precision medicine to be practiced (69). While evidence-based medicine emphasizes the importance of evidence quality in guiding practice guidelines, precision-based medicine goes a step further by identifying the relevant patient(s). Precision medicine uses molecular evidence to identify drug targets and patient responses to drugs (70). A classic example of this is the EGFR gene in metastatic non-small cell lung cancer. EGFR inhibitors such as gefitinib and erlotinib when administered to patients positive for either rs121434568 (T) or rs121434569 (C). SNP are predicted to show decreased response and lower progression-free survival time (71). Precision medicine has opened up basket trials that are being performed in a tissue agnostic manner to treat cancer based on the molecular landscape of patients. Prioritization of genes of interest based on their level of association evidence is critical for maximizing the utility of PGx big data by directing efforts to the most promising gene candidates (72). Currently, collaborative efforts are ongoing between various genomic institutes worldwide to suggest and validate an innovative model for testing an optimal gene list (73). An example of this in the United States is the cooperation between the Implementing GeNomics In pracTiCe (IGNITE), Clinical Trials Network (http://www.ignite-genomics.org/) with the electronic medical records and genomics (eMERGE), Network (https://emerge-network.org/). Similarly, a large study was conducted on 8,000 subjects from seven European countries (the Netherlands, Spain, UK, Italy, Austria, Greece, and Slovenia) in an attempt to assess the safety level and cost-effectiveness of testing a specified gene panel in a PGx program called Ubiquitous (U-PGx) (74). We believe that our shortlisted panel discussed in this study may contribute to tailoring the most appropriate pharmacogenes for preemptive testing.

## Conclusion

This study highlighted 50 genes with strong evidence of association with variable responses to 152 drugs. The suggested genes fit the top two levels of evidence criteria of at least one of the indicated scoring databases: CPIC, DPWG, and PharmGKB. The genes CYP2D6, CYP2C9, CYP2C19, G6PD, HLA-B, SLCO1B1, RYR1, CACNA1S, MT-RNR1, and INFL4 influence a wide range of drug therapies. Prioritizing the tested pharmacogenes would be helpful in easing the PGx implementation process.

## Data availability statement

The original contributions presented in the study are included in the article/Supplementary material, further inquiries can be directed to the corresponding author.

## Ethics statement

The study was approved by the Institutional Review Board (IRB) at King Abdullah International Medical Research Center (KAIMRC) (NRC21R/394/09). Written informed consent from the patients/participants or patients/participants legal guardian/next of kin was not required to participate in this study in accordance with the national legislation and the institutional requirements.

## Author contributions

MAls and MAly conceived and conceptualized the work. MAz prepared the supplementary materials. SA shared in manuscript preparation. All authors have agreed to all of the submitted materials.

## Conflict of interest

The authors declare that the research was conducted in the absence of any commercial or financial relationships that could be construed as a potential conflict of interest.

## Publisher's note

All claims expressed in this article are solely those of the authors and do not necessarily represent those of their affiliated organizations, or those of the publisher, the editors and the reviewers. Any product that may be evaluated in this article, or claim that may be made by its manufacturer, is not guaranteed or endorsed by the publisher.

## Abbreviations

ACE: Angiotensin-Converting Enzyme
ADRs: Adverse Drug Reactions
ACMG: American College of Medical Genetics and Genomics
CPNDS: Canadian Pharmacogenomics Network for Drug Safety
CPIC: Clinical Pharmacogenetics Implementation Consortium
DPWG: Dutch Pharmacogenetics Working Group
EMA: European Medicine Agency
eMERGE: electronic Medical Records and Genomics
HCSC: Canadian health agency; Health Canada (Santé Canada)
HCV: Hepatitis C Virus
HLA: Human Leukocyte Antigen
IGNITE: Implementing GeNomics In practice
JAK-STAT: Janus kinase-signal transducer and activator of transcription
MHC: Major Histocompatibility Complex
NIH: National Institutes of Health
NSAIDs: Non-Steroidal Anti-Inflammatory Drugs
PGX: Pharmacogenomic (s)
PharmGKB: Pharmacogenomics Knowledge web-Base
PMDA: Pharmaceuticals and Medical Devices Agency
RNPGx: French National Network of Pharmacogenetics
SARS-CoV-2: Severe Acute Respiratory Syndrome Coronavirus 2
US: United States
USFDA: United States Food and Drug Administration
VIP: Very Important Pharmacogenes.
