# Supplementary material for: Pharmacogenes that demonstrate high association evidence according to CPIC, DPWG, and PharmGKB
Source: Front Med (Lausanne). 2022 Oct 25;9:1001876. doi: 10.3389/fmed.2022.1001876 (PMC9640910; doi:10.3389/fmed.2022.1001876)
Supplement: Supplementary file 1 [file Table_1.pdf]

**Supplementary Table:** Official full names of pharmacogenes, ontology, biological function, and gene ID as seen in the National Center for Biotechnology Information (NCBI) database.

| Symbol | Gene Name | Gene Ontology | Biological function | Gene ID as seen in NCBI |
|---|---|---|---|---|
| **ABCG2** | ATP binding cassette subfamily G member 2 (Junior blood group) | Plasma Membrane | transporter | 9429 |
| **ACE** | angiotensin I converting enzyme | Plasma Membrane | enzyme | 1636 |
| **ADD1** | adducin 1 | Plasma Membrane | transporter | 118 |
| **ADRB2** | adrenoceptor beta 2 | Plasma Membrane | receptor | 154 |
| **APOE** | apolipoprotein E | Extracellular Space | transporter | 348 |
| **ATIC** | 5-aminoimidazole-4-carboxamide ribonucleotide formyltransferase/IMP cyclohydrolase | Cytoplasm | enzyme | 471 |
| **CACNA1S** | calcium voltage-gated channel subunit alpha1 S | Plasma Membrane | ion channel | 779 |
| **CES1** | carboxylesterase 1 | Cytoplasm | enzyme | 1066 |
| **CFTR** | CF transmembrane conductance regulator | Plasma Membrane | ion channel | 1080 |
| **CHRNA5** | cholinergic receptor nicotinic alpha 5 subunit | Plasma Membrane | receptor | 1138 |
| **CPS1** | carbamoyl-phosphate synthase 1 | Cytoplasm | enzyme | 1373 |
| **CYP2A6** | cytochrome P450 family 2 subfamily A member 6 | Cytoplasm | enzyme | 1548 |
| **CYP2B6** | cytochrome P450 family 2 subfamily B member 6 | Cytoplasm | enzyme | 1555 |
| **CYP2C9** | cytochrome P450 family 2 subfamily C member 9 | Cytoplasm | enzyme | 1559 |
| **CYP2C19** | cytochrome P450 family 2 subfamily C member 19 | Cytoplasm | enzyme | 1557 |
| **CYP2D6** | cytochrome P450 family 2 subfamily D member 6 | Cytoplasm | enzyme | 1565 |
| **CYP3A4** | cytochrome P450 family 3 subfamily A member 4 | Cytoplasm | enzyme | 1576 |
| **CYP3A5** | cytochrome P450 family 3 subfamily A member 5 | Cytoplasm | enzyme | 1577 |
| **CYP4F2** | cytochrome P450 family 4 subfamily F member 2 | Cytoplasm | enzyme | 8529 |
| **DPYD** | dihydropyrimidine dehydrogenase | Cytoplasm | enzyme | 1806 |

| | | | | |
|---|---|---|---|---|
| **EGFR** | epidermal growth factor receptor | Plasma Membrane | receptor | 1956 |
| **FCGR3A** | Fc fragment of IgG receptor IIIa | Plasma Membrane | receptor | 2214 |
| **FVL (F5)** | Factor V Leiden | Extracellular Space | coagulation cofactor | 2153 |
| **GBA** | glucosylceramidase beta | Cytoplasm | enzyme | 2629 |
| **G6PD** | glucose-6-phosphate dehydrogenase | Cytoplasm | enzyme | 2539 |
| **HLA-A** | major histocompatibility complex, class I, A | Plasma Membrane | antigen presentation | 3105 |
| **HLA-B** | major histocompatibility complex, class I, B | Plasma Membrane | antigen presentation | 3106 |
| **HLA-C** | major histocompatibility complex, class I, C | Plasma Membrane | antigen presentation | 3107 |
| **HLA- DPB1** | major histocompatibility complex, class II, DP beta 1 | Plasma membrane | antigen presentation | 3115 |
| **HLA-DRB1** | major histocompatibility complex, class II, DR beta 1 | Plasma membrane | antigen presentation | 3123 |
| **HPRT1** | hypoxanthine phosphoribosyltransferase 1 | Cytoplasm | enzyme | 3251 |
| **IFNL3 (IL28B)** | interferon lambda 3 | Extracellular Space | cytokine | 282617 |
| **IFNL4** | interferon lambda 4 | Cytoplasm | cytokine | 101180976 |
| **ITPA** | inosine triphosphatase | Cytoplasm | enzyme | 3704 |
| **KIF6** | kinesin family member 6 | Nucleus | microtubule-based movement | 221458 |
| **MTHFR** | methylenetetrahydrofolate reductase | Cytoplasm | enzyme | 4524 |
| **MT-RNR1** | mitochondrially encoded 12S RNA | Cytoplasm | insulin sensitivity regulator | 4549 |
| **NAGS** | N-acetylglutamate synthase | Cytoplasm | enzyme | 162417 |
| **NAT2** | N-acetyltransferase 2 | Cytoplasm | enzyme | 10 |
| **NUDT15** | nudix hydrolase 15 | Cytoplasm | enzyme | 55270 |
| **OTC** | ornithine carbamoyltransferase | Cytoplasm | enzyme | 5009 |
| **POLG** | DNA polymerase gamma, catalytic subunit | Cytoplasm | enzyme | 5428 |
| **RYR1** | ryanodine receptor 1 | Cytoplasm | ion channel | 6261 |
| **SCN1A** | sodium voltage-gated channel alpha subunit 1 | Plasma Membrane | ion channel | 6323 |
| **SLC19A1** | solute carrier family 19 member 1 | Plasma Membrane | transporter | 6573 |
| **SLCO1B1** | solute carrier organic anion transporter family member 1B1 | Plasma Membrane | transporter | 10599 |
| **TNF-α** | tumor necrosis factor-alpha | Intracellular | cytokine | 7124 |

| | | | | |
|---|---|---|---|---|
| | | Space | | |
| **TPMT** | thiopurine S-methyltransferase | Cytoplasm | enzyme | 7172 |
| **UGT1A1** | UDP glucuronosyltransferase family 1 member A1 | Cytoplasm | enzyme | 54658 |
| **VKORC1** | vitamin K epoxide reductase complex subunit 1 | Cytoplasm | enzyme | 79001 |
